# Identification of Homozygous Regions With Adverse Effects on the Five Economic Traits of Duroc Pigs

**DOI:** 10.3389/fvets.2022.855933

**Published:** 2022-04-28

**Authors:** Shiyuan Wang, Jie Yang, Guixin Li, Rongrong Ding, Zhanwei Zhuang, Donglin Ruan, Jie Wu, Huaqiang Yang, Enqin Zheng, Gengyuan Cai, Xiaopeng Wang, Zhenfang Wu

**Affiliations:** ^1^College of Animal Science and National Engineering Research Center for Breeding Swine Industry, South China Agricultural University, Guangzhou, China; ^2^Guangdong Provincial Laboratory of Lingnan Modern Agricultural Science and Technology, Guangzhou, China

**Keywords:** Duroc pigs, runs of homozygosity, economic traits, adverse effects, breeding

## Abstract

Runs of homozygosity (ROH) are widely used to estimate genomic inbreeding, which is linked to inbreeding depression on phenotypes. However, the adverse effects of specific homozygous regions on phenotypic characteristics are rarely studied in livestock. In this study, the 50 K SNP data of 3,770 S21 Duroc (American origin) and 2,096 S22 Duroc (Canadian origin) pigs were used to investigate the harmful ROH regions on five economic traits. The results showed that the two Duroc lines had different numbers and distributions of unfavorable ROHs, which may be related to the different selection directions and intensities between the two lines. A total of 114 and 58 ROH segments were found with significant adverse effects on the economic traits of S21 and S22 pigs, respectively. Serval pleiotropic ROHs were detected to reduce two or multiple phenotypic performances in two Duroc populations. Candidate genes in these shared regions were mainly related to growth, fertility, immunity, and fat deposition. We also observed that some ROH genotypes may cause opposite effects on different traits. This study not only enhances our understanding of the adverse effects of ROH on phenotypes, but also indicates that ROH information could be incorporated into breeding programs to estimate and control the detrimental effects of homozygous regions.

## Introduction

Runs of homozygosity (ROH) are a continuous segment of homozygous genotype in the genome of diploid organisms, which arise when two copies of the same ancestral haplotype are gathered together in one individual (1). Long haplotype fragments are derived from a closer common ancestor, whereas short haplotype fragments are derived from a distantly related common ancestor (2). The formation of ROH patterns on the genome can be influenced by many factors, including inbreeding, genetic drift, mating system, selection intensity, effective population size, population structure, and genetic linkage (1, 3, 4). The development history of inbreeding can also be inferred, because the fewer the generation, the less likely the ROH fragment will be interrupted by recombination events (5, 6). Therefore, ROH can reveal valuable information about the genetic background of an animal population. In 1999, Broman and Weber (3) first reported the long homozygous fragment and found that the length of homozygous fragment was related to human disease. The widespread application of single nucleotide polymorphism (SNP) chips and whole-genome resequencing data provides a great opportunity for the research of ROH in livestock. For livestock genomes, the detected ROH can be used to assess the inbreeding degree (7–9), infer population inbreeding and evolutionary history (5, 10, 11), identify positive selection (11, 12) and deleterious mutations (13–15), estimate genetic diversity and conduct the conservation of genetic resources (16–18), and design animal breeding schemes (13, 19). ROH are considered to be a better estimate of inbreeding than pedigree (4). High inbreeding levels usually lead to inbreeding depression, which is related to reducing the fitness, reproduction, and production performances in livestock (20). However, few people paid attention to the adverse effects of ROH on phenotypic traits. Recently, the detrimental effects of ROH on the phenotype have also begun to be explored, because the rational use of this unfavorable haplotype information in the breeding process can improve genetic progress to a certain extent. For example, Howard et al. (21) developed an algorithm of Unfavorable Haplotype Finder software (*Haplofinder*) that can detect unfavorable ROH genotypes associated with phenotypes. Then, Martikainen et al. (22) used *Haplofinder* software to identify ROH fragments that were not conducive to the fertility and milk production of Finnish Ayrshire cattle; Makanjuola et al. (23) used same software to uncover unique ROH segments with adverse effects on fertility and production traits in Canadian Holstein cattle.

Duroc pig is one of the most popular commercial breeds worldwide. Recently, intensive selection in the Duroc population has resulted in significant genetic gains in multiple economic traits of interest. Duroc pigs have become the basis for many mixed-breed commercial lean boars due to their excellent characteristics in terms of growth, feed conversion efficiency, physique, carcass, and meat quality (24). For example, Duroc × (Landrace × Yorkshire) (DLY) commercial pigs dominated the pork market in China. The strong selection of superior individuals reduces phenotypic variability and reshapes the ROH pattern in the population genome (4). Selection could also enhance the occurrence frequency of deleterious mutations in the ROH region, thereby increasing the possibility of recessive diseases (4, 14), which will have unfavorable effects on economic traits. The current studies on the ROH of Duroc pigs were mainly to evaluate the levels of inbreeding and to detect phenotype-related genes in ROH hotspots that were putatively under positive selection (25–28). However, the adverse effects of ROH on the economic traits of Duroc pigs have not been studied. Here, we genotyped 3,770 S21 Duroc (American origin) and 2,096 S22 Duroc (Canadian origin) pigs using 50 K SNP chip, and the objective of our study was to identify the unique ROHs that negatively affected five important economic traits.

## Materials and Methods

### Ethics Statement

The experimental procedures used in this study met the guidelines of the Animal Care and Use Committee of the South China Agricultural University (SCAU) (Guangzhou, China). The Animal Care and Use Committee of the SCAU approved all animal experiments described in this study.

### Data Management and Quality Control

In this study, a total of 5,866 pigs were sampled from the Wen's Foodstuff Group Co., Ltd. (Guangdong, China). To minimize the impact of nongenetic factors, all pigs were subjected to the same growth and feeding conditions during the fattening period from 30- to 100-kg live weight. Of the 5,866 pigs, 3,770 S21 pigs were born between 2013 and 2017, and 2,096 S22 pigs were born between 2016 and 2017. A total of five economic traits were recorded including average daily gain (ADG) at 100 kg, backfat thickness (BFT), loin muscle area (LMA), lean meat percentage at 100 kg (LMP), and total teat number (TTN) (Supplementary Table S1). The two Duroc lines had moderate genetic differentiation without sampling error, and the detailed descriptions of samples and phenotypes can be found in our previous papers (29–32).

Genomic DNA was isolated from ear samples using the standard phenol-chloroform method. Genotyping was performed using the GeneSeek Porcine 50 K SNP Chip containing 50,703 SNP markers (29). PLINK v1.90 software (33) was used to filter the genotyping data with the parameters of call rates > 0.90, minor allele frequencies > 0.01, and *p* > 10^−6^ for Hardy–Weinberg equilibrium test. SNPs without position information and those on the sex chromosomes and duplicates were also eliminated. Finally, 39,416 informative SNPs of 3,770 individuals in S21 population and 35,850 informative SNPs of 2,096 individuals in S22 population were retained for subsequent analyses.

### Correlation Analyses Among ROH and Five Economic Traits

To evaluate the relationships between ROH and economic traits, the total ROH length of each individual was retrieved from our previous study (34). Put simply, ROH were detected using the R *detectRUNS* package v0.9.6 (35) with consecutive method and allowed each segment to have one heterozygote and one genotype to be missed, a minimum of 15 SNPs and 500 kb in a run. Then, Pearson correlation analysis was performed between total ROH length and economic traits using the R package *corr.test*. In the meantime, the correlation matrix in R was also used to conduct correlation analyses among the five economic traits of S21 and S22 pigs. The R package *corrplot* was used to visualize the correlation and significance values.

### Unfavorable ROH Detection

*Haplofinder* (21) software was used to detect genotypes within ROHs, which had unfavorable effects on the economic traits in two Duroc lines. Howard et al. (21) gave a complete description of the software algorithm. This algorithm consists of three steps to identify haplotypes. First step, a sliding window of decreasing marker size was used to identify ROH segments with adverse effects on phenotypes. The minimum frequency of each unique ROH genotype was set as 0.75% (default setting). The ROH segments that have no heterozygous SNPs and exceed the minimum frequency of 0.75% are classified into the ROH category, whereas other segments are classified into the non-ROH category. The phenotypic mean of each unique ROH genotype was then estimated using all individuals carrying the unique ROH genotype. In *Haplofinder* parameter settings, the parameter “UNFAV” is used to indicate the direction of haplotype settings, such as, when this parameter is set to “low,” haplotypes with an average phenotypic value lower than the cutoff value are considered unfavorable haplotypes. We set the “UNFAV” of ADG, LMA, LMP, and TTN to “low,” and BFT to “high”. Depending on the unfavorable direction, windows containing ROH genotypes with phenotypic means above or below a user-defined threshold are stored for further analysis. A smaller sliding window was used to treat the average ROH genotype as a marker for the entire ROH genotype. After sorting, during the 5-SNP interval, the window has been reduced from 60 to 15 SNPs, and the cutoff threshold for the average phenotypic value was estimated by using 1,000 permutations of random genomic regions. Further testing is carried out in the next step.

Next, a linear mixed model was used to test the significance of each genotype found in the previous step. The default linear mixed model of the software is as follows:


y=Xb+Za+Wpe+e,

where *y* is a vector of the target economic traits (ADG, BFT, LMA, LMP, and TTN), *b* is a vector of fixed effects (environmental effects and the effect of the ROH genotype in a given window), *a* means a random additive genetic effects, *e* is a vector of random residuals, and X, Z, and W are incidence matrices that relate b, a, and pe to *y*, respectively. The random additive genetic effect is assumed to be normally distributed with N(0, A$\sigma_{\text{a}}^{2}$), where A indicates the pedigree additive relationship matrix and $\sigma_{\text{a}}^{2}$ is the additive genetic variance. The random permanent environmental and residual effects are assumed to be distributed ~N(0, I$\sigma_{\text{pe}}^{2}$) and ~N(0, I $\sigma_{\text{e}}^{2}$), respectively, where I is an identity matrix. Based on the null hypothesis of no ROH effect, the variance components of this implementation were assumed to be fixed across windows. Each window and each individual ROH segment are divided into two groups: one group including animals with the unfavorable ROH segment, and the other group containing animals without the unfavorable ROH segment in the window tested. A one-sided *t*-test was then carried out on these groups, which only considered the adverse direction of the ROH genotypes, and thus, animals with non-ROHs were assumed to be the baseline for comparison with animals carrying ROHs. In this way, ROHs with significant effects on traits were identified. In the last step, the software would remove the nested windows.

Since Bonferroni correction is a relatively strict criterion for multiple testing (31, 36), a false discovery rate (FDR) = 0.01 was used in this study to determine the threshold *p*-value of significant ROH regions. Considering that recent inbreeding has more adverse effects compared to ancient inbreeding (37), we then retained significantly unfavorable ROHs with a minimum of 50 SNPs following the previous literature (23), because such a window size captures the more recent inbreeding (38). R package *ggplot* was employed to plot the candidate ROHs of the phenotypic traits in two Duroc lines. The overlapping regions displayed in the plots were integrated into the shared ROH fragments through BEDtools software (39). Finally, gene annotation file was accessed from Ensembl database (*Sus scrofa 11.1*, http://asia.ensembl.org/), and gene annotation for these shared candidate ROH fragments was analyzed using R package *GALLO* (40).

## Results

### Relationships Among ROH and Five Economic Traits

Pearson correlation test was used to infer the relationships among total ROH length and five economic traits. According to the classification of correlation coefficients (41), the results showed that total ROH length had no correlation (*r* < 0.1) with five economic traits in two Duroc lines (Supplementary Figure S1). This revealed that the inbreeding control of two populations was successful, and there was no inbreeding depression for economic traits at the population level. Nevertheless, we observed that total ROH length had potentially negative effects on some economic traits, such as ADG in S21 (*r* = −0.081, *p* = 7.39 × 10^−7^) and S22 (*r* = −0.051, *p* = 0.02) pigs. We also found that several pairs of traits had significant correlations in two Duroc lines, indicating that these traits possibly harbored similar or opposite genetic basics.

### Unfavorable ROH Detection

#### Assessment of Unfavorable ROHs

**Figure 1:**
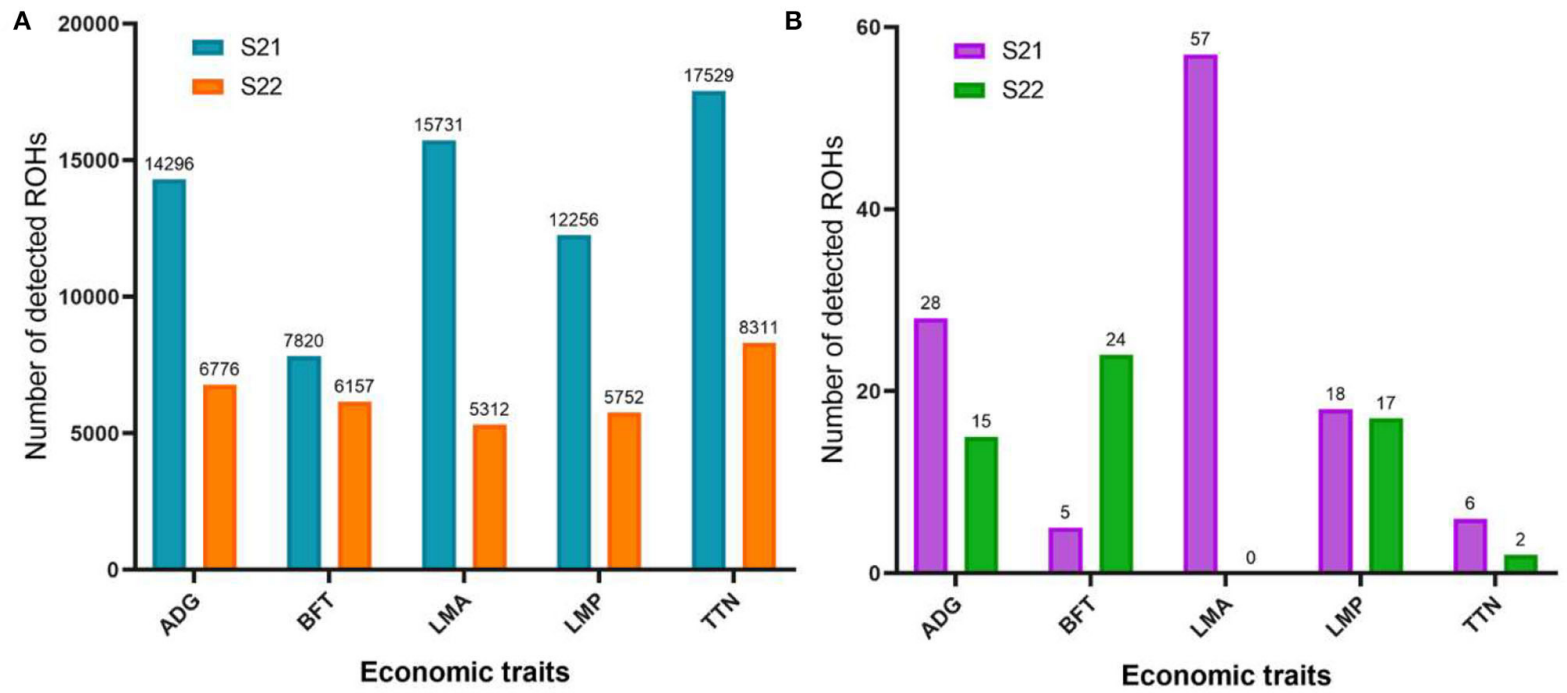
Number of unfavorable ROHs detected in two Duroc populations. **(A)** Total number of detected unfavorable ROHs. **(B)** Number of significantly unfavorable ROHs based on FDR = 0.1 and at least 50 SNPs. S21, Duroc pigs of American origin; S22, Duroc pigs of Canadian origin; ADG, average daily gain at 100 kg; BFT, backfat thickness; LMA, loin muscle area; LMP, lean meat percentage at 100 kg; and TTN, total teat number.

In this study, ROH segments with unfavorable effects on five important economic traits were identified in two Duroc lines. The total number of unfavorable ROH regions ranged from 7,820 to 17,529 and 5,312 to 8,311 on the five economic traits of S21 and S22 pigs (Figure 1A), respectively. The number of unfavorable ROHs on TTN was the largest in two Duroc lines (Figure 1A). The FDR = 0.01 and at least 50 SNPs were used to define the threshold for detecting significantly unfavorable ROHs. The number of significantly unfavorable ROH fragments for each trait was different in two Duroc lines, and not all autosomes contained ROHs that were harmful to all traits (Table 1, Supplementary Tables S2, S3). For S21 pigs, the number of significantly adverse ROH regions ranged from 5 in BFT to 57 in LMA (Figure 1B), and the average length of unfavorable ROHs ranged from 1.93 Mb in TTN to 3.32 Mb in ADG. In addition, animals carrying these ROHs had an average of 8.18 g, 1.00 cm^2^, 0.29%, 0.3 less ADG, LMA, LMP, and TTN and 0.51 mm more BFT, respectively, than the animals without these genotypes (Table 1). In comparison, a number of significantly adverse ROH fragments in S22 pigs were 15, 24, 17, and 2 for ADG, BFT, LMP, and TTN, respectively, whereas no ROHs were significantly associated with LMA (Figure 1B). The average length of adverse ROHs ranged from 2.70 Mb in BFT to 3.66 Mb in ADG. Individuals carrying these unfavorable ROH segments reduced an average of 16.46 g ADG, 0.63% LMP, and 0.49 TTN and increased an average of 0.99 mm BFT, respectively, in contrast to the individuals with non-ROH segments (Table 1). Similar to the pattern of total adverse ROH numbers, S21 pigs had more significant candidate ROHs than S22 pigs, expect for BFT trait (Figure 1B).

**Table 1:** Information of unique ROHs with significantly adverse effects on five economic traits.

**Traits**	**N_chr_**	**Average number of SNPs**	**Average (Mb)**	**Minimum (Mb)**	**Maximum (Mb)**	**ROH effect**
S21 population
ADG (g)	14	51.29	3.32	1.20	9.27	−8.18
BFT (mm)	5	51	2.34	1.06	3.83	0.51
LMA (cm^2^)	15	51	2.93	1.04	12.43	−1.00
LMP (%)	7	51.17	2.54	1.72	3.18	−0.29
TTN	5	50.5	1.93	1.27	2.87	−0.30
S22 population
ADG (g)	10	51.67	3.66	1.62	8.13	−16.46
BFT (mm)	10	51.13	2.70	1.56	9.02	0.99
LMP (%)	9	50.88	3.19	1.79	7.18	−0.63
TTN	2	51	3.58	3.10	4.07	−0.49

*S21, Duroc pigs of American origin; S22, Duroc pigs of Canadian origin; ADG, average daily gain at 100 kg; BFT, backfat thickness; LMA, loin muscle area; LMP, lean meat percentage at 100 kg; TTN, total teat number; N_chr_, number of chromosomes carrying adverse ROHs; ROH effect, average effect of unique ROH compared to non-ROH category*.

#### Shared Unfavorable ROHs Between Traits in Two Duroc Lines

**Figure 2:**
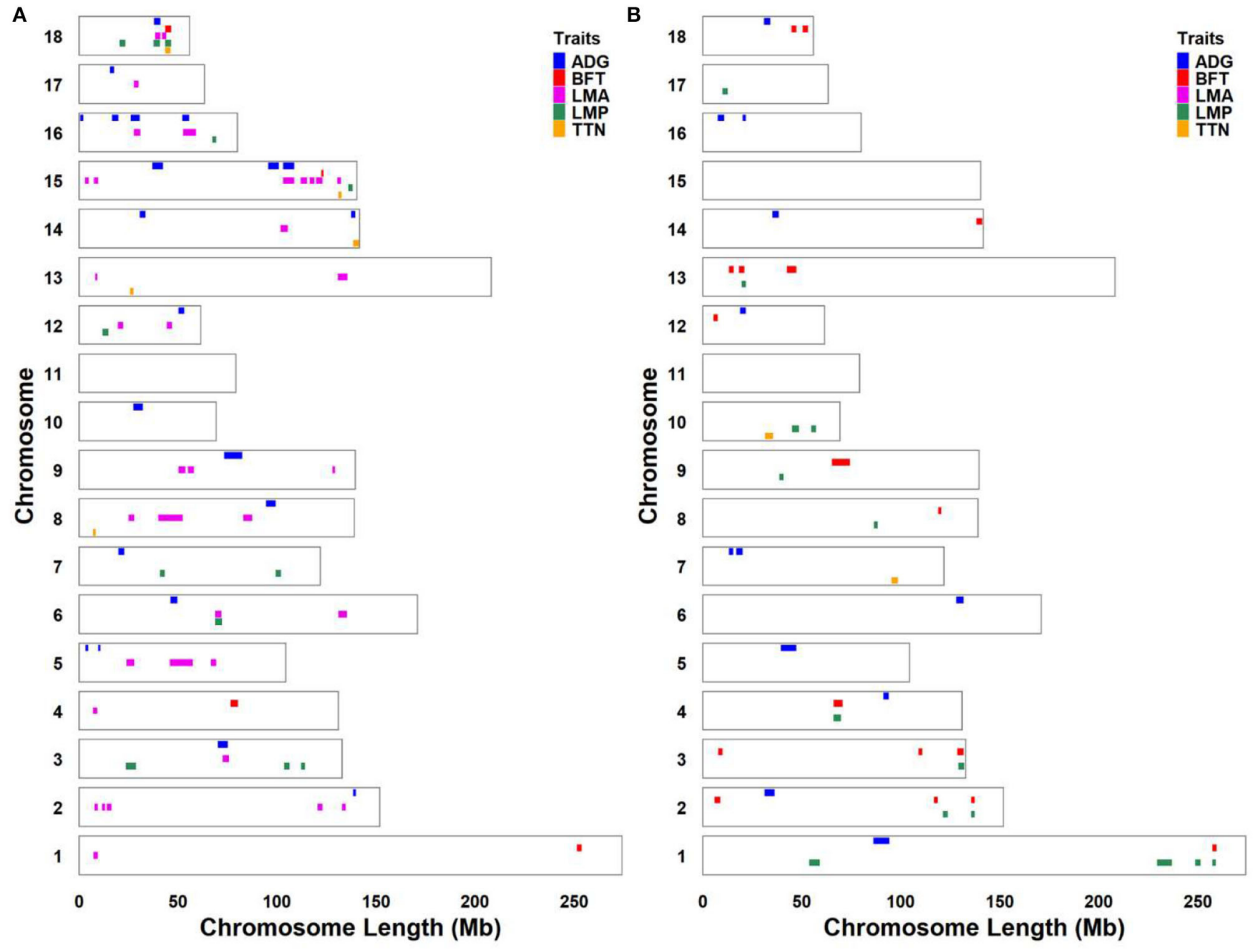
Genomic distribution of significantly unfavorable ROH regions for economic traits. **(A)** S21 population. **(B)** S22 population. S21, Duroc pigs of American origin; S22, Duroc pigs of Canadian origin; ADG, average daily gain at 100 kg; BFT, backfat thickness; LMA, loin muscle area; LMP, lean meat percentage at 100 kg; and TTN, total teat number.

**Figure 3:**
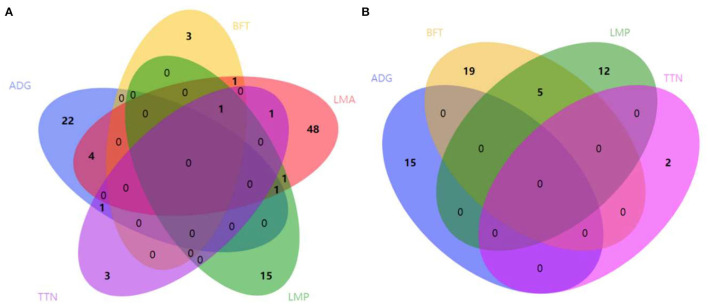
Number of pleiotropic ROHs across economic traits. **(A)** S21 population. **(B)** S22 population. S21, Duroc pigs of American origin; S22, Duroc pigs of Canadian origin; ADG, average daily gain at 100 kg; BFT, backfat thickness; LMA, loin muscle area; LMP, lean meat percentage at 100 kg; and TTN, total teat number. Venn diagrams are plotted using the web server (http://www.ehbio.com/test/venn/).

**Figure 4:**
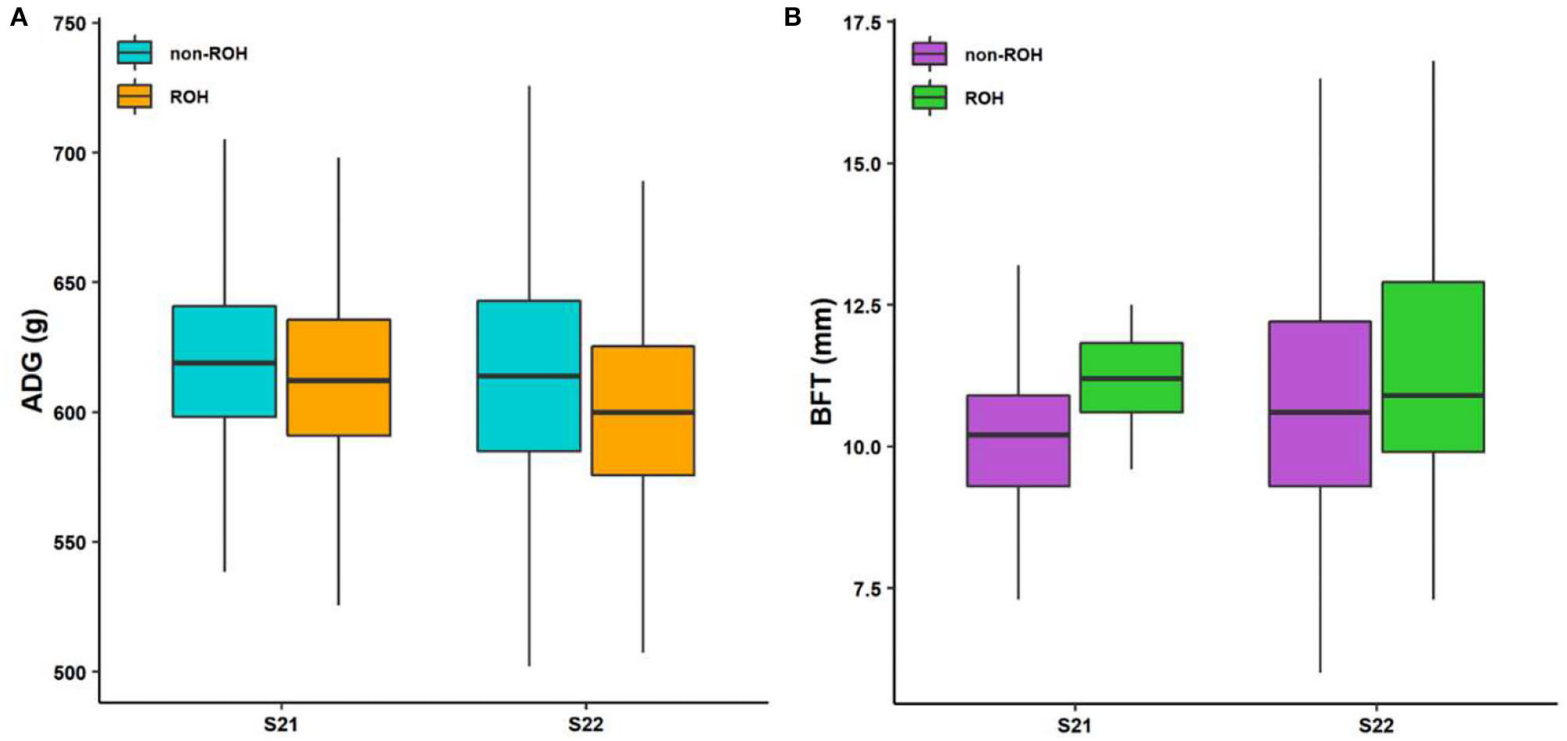
Boxplot of the phenotypic values between individuals with ROH and non-ROH. **(A)** ROH with adverse effect on ADG. **(B)** ROH with adverse effect on BFT. S21, Duroc pigs of American origin; S22, Duroc pigs of Canadian origin; ADG, average daily gain at 100 kg; and BFT, backfat thickness.

Considering that some traits had genetic relationships with each other, we identified a lot of pleiotropic ROHs across these economic traits (Table 2). For S21 pigs, a total of 10 pleiotropic ROH regions were detected on chromosomes (SSC) 3, 6, 14, 15, 16, and 18 (Figure 2A and Table 2). Then, four, one, one, one, and one unique ROHs were overlapped between ADG and LMA, ADG and TTN, LMA and LMP, LMA and TTN, and BFT and LMA (Figure 3A), respectively. Interestingly, two genotypes were observed on SSC18 with adverse effects on three and four traits, including 38.51–40.79 Mb for ADG, LMA and LMP, and 43.60–44.00 Mb for BFT, LMA, LMP, and TTN. In comparison, five pleiotropic genotypes were found on SSC1, 2, 3, 4, and 13 in S22 pigs (Figure 2B and Table 2). These five segments all significantly affected the BFT and LMP traits (Figure 3B). For two populations, two overlapping ROH genotypes (SSC7: 20.05–20.30 Mb and SSC18: 44.82–46.52 Mb) were detected to affect ADG and BFT traits, respectively (Table 2 and Figure 4). Compared to pigs without these genotypes, pigs with the genotype on SSC7 had a difference of −5.56 and −10.87 g in ADG (Figure 4A), whereas pigs with the genotype on SSC18 had 0.82 and 0.73 mm more BFT in S21 and S22 populations (Figure 4B), respectively.

**Table 2:** Unfavorable ROHs shared by economic traits and two Duroc populations.

**Chr**	**Position (Mb)**	**Trait**	**ROH effect**	**–log10(P)**	**Gene**
*S21 population*
3	72.57–75.25	ADG	−6.22	3.20	*ANTXR1, GKN1, PPP3R1, PNO1*
		LMA	−0.98	3.94	
6	68.83–71.88	LMA	−0.63	3.88	*SLC2A5, H6PD, TARDBP, MTOR*
		LMP	−0.22	3.82	
14	138.54–139.72	ADG	−9.44	2.80	*/*
		TTN	−0.30	3.79	
15	103.16–108.85	ADG	−7.59	3.11	*CASP8, BMPR2, ICA1L, ABI2, CD28, CTLA4, ICOS*
		LMA	−1.14	4.36	
15	122.54–122.98	BFT	0.31	3.08	*/*
		LMA	−1.16	4.15	
15	130.95–132.31	LMA	−0.51	2.76	*GPR55*
		TTN	−0.16	3.98	
16	27.62–30.61	ADG	−4.98	4.67	*FGF10*
		LMA	−0.74	6.26	
16	52.74–55.66	ADG	−8.13	3.04	*FGF18, DOCK2*
		LMA	−1.09	3.27	
18	38.51–40.79	ADG	−6.77	3.67	*DPY19L2, BMPER, BBS9*
		LMA	−0.82	3.14	
		LMP	−0.22	3.92	
18	43.60–44.00	BFT	0.82	3.20	*CPVL, CREB5*
		LMA	−1.75	4.80	
		LMP	−0.55	3.82	
		TTN	−0.27	3.44	
*S22 population*
1	257.44–259.26	BFT	1.75	5.67	*TLR4*
		LMP	−1.47	6.24	
2	135.69–137.48	BFT	1.54	4.97	*PPP2CA, UBE2B, CATSPER3, PITX1*
		LMP	−0.91	4.38	
3	129.41–131.83	BFT	0.77	4.93	*SOX11*
		LMP	−0.39	3.79	
4	66.50–69.75	BFT	0.67	4.59	*CRH, CYP7B1*
		LMP	−0.44	4.76	
13	19.80–21.12	BFT	1.59	3.02	*ARPP21*
		LMP	−0.35	3.15	
*S21 overlapped with S22*
7	20.05–20.30	ADG^a^	−5.56	3.12	*CARMIL1*
		ADG^b^	−10.87	3.27	
18	44.82–46.52	BFT^a^	0.82	3.20	*JAZF1, HOXA1, HOXA2, HOXA3, HOXA5, HOXA7, HOXA10, HOXA11, HOXA13*
		BFT^b^	0.73	3.46	

a *S21 pigs*.

b *S22 pigs*.

*S21, Duroc pigs of American origin; S22, Duroc pigs of Canadian origin; Chr, chromosome; ADG, average daily gain at 100 kg; AGE, days to 100 kg; BFT, backfat thickness; LMA, loin muscle area; LMP, lean meat percentage at 100 kg; TTN, total teat number; ROH effect, effect of identified ROH in comparison to the non-ROH category; gene, some important functional genes related to traits in the fragment. The units of the phenotypes are in Table 1*.

### Candidate Genes in Unfavorable ROHs

To reveal the genetic basis of unfavorable ROHs on the important economic traits of Duroc pigs, we annotated candidate genes within significantly adverse ROHs and analyzed the biological functions of these genes (Table 2 and Supplementary Table S4). For S21 population, in five overlapping ROH regions between ADG and LMA, seven genes (*PPP3R1, PNO1, CASP8, CD28, CTLA4, ICOS*, and *DOCK2*) were related to the immune response; three genes (*FGF10, ANTXR1*, and *FGF18*) were associated with growth and skeletal development; two genes were involved in gametogenesis (*ICA1L*) and embryonic development (*BMPR2*); and one gene (*GKN1*) was relevant to fat deposition. In the common ROH region between ADG and TTN, gene *DGRX3* was related to mammary gland development. In the overlapping ROH segment between LMA and LMP, genes *SLC2A5, H6PD, TARDBP*, and *MTOR* were associated with nutrient absorption, muscle development, fat deposition, and embryonic development, respectively. *GPPR55* gene involved in adipogenesis was detected in the identical ROH genotype of LMA and TTN. We also observed that three genes related to embryonic development, growth, and skeletal development were located on the overlapping ROH region among ADG, LMA, and LMP. In addition, *CPVL* and *CREB5* genes associated with immunity and adipocyte differentiation, respectively, were detected in the overlapping ROH among BFT, LMA, LMP, and TTN traits. For S22 population, in five overlapping ROHs between BFT and LMP, three genes were related to lipid metabolism (*CRH*), fatty acid composition (*CYP7B1*), and obesity (*TLR4*); two genes (*PITX1* and *ARPP21*) were associated with muscle development; and four genes were involved in spermatogenesis (*CATSPER3, PPP2CA* and *UBE2B*) and embryonic development (*SOX11*). Moreover, we detected that two overlapping ROHs significantly affected the ADG and BFT traits in two Duroc lines, respectively. *CARMIL1* gene related to growth was located on the unfavorable ROH for ADG. *JAZF1* and eight homeobox genes (e.g., *HOXA1, HOXA2, HOXA3, HOXA5, HOXA7, HOXA10, HOXA11*, and *HOXA13*) associated with lipid accumulation were located on the adverse ROH for BFT.

## Discussion

In this work, we used whole-genome 50 K SNP data from two Duroc pig lines to investigate the effects of ROH fragments on five important economic traits. Intensive selection possibly increases the unfavorable alleles due to genetic hitchhiking (42) and then makes harmful effects on the phenotypes. A previous study reported that deleterious mutations are more enriched in ROH regions, especially long ROH regions (14). Commercial livestock has recently experienced intense artificial selection to improve target traits, which may accumulate a large number of long ROH genotypes. These ROHs are not all stacks of favorable alleles, and some of them may also be harmful to the phenotypes (inbreeding depression) (21). During the breeding process of commercial lines, a large amount of available genetic data and accurate and comprehensive phenotypic information were produced. These provide excellent experimental materials for estimating the association between ROH and phenotypes. Considering that Howard et al. (21), Martikainen et al. (22), and Makanjuola et al. (23) successfully detected unique ROH genotypes with adverse effects on the production and fertility traits of Landrace and Large White pigs, Finnish Ayrshire cattle, and Canadian Holsteins cow, respectively. We used the same *HaploFinder* software to identify unfavorable ROH genotypes within traits and across multiple traits.

The results showed that S21 had more total unfavorable ROHs on five traits than that of S22 population. According to previous description (23), stringent criteria with a significant threshold of FDR = 0.01 and at least 50 SNPs in a ROH were used to identify significantly unfavorable ROH regions in two Duroc lines. Although these options may ignore some valuable genotypes, the regions we detected were expected to be the most deleterious to the phenotypes and were formed in recent generations. The number of significantly adverse ROHs on the economic traits (except for BFT) of S21 was larger than that of S22 pigs. The population inbreeding coefficient may be positively correlated with the number of adverse ROHs; however, our previous study found that S21 pigs had lower inbreeding levels than S22 pigs (43). On the one hand, this may be due to the sample bias, whereas on the other hand, the difference may result from the two Duroc lines experiencing different selection directions and intensities. The later hypothesis was in line with our previous studies that S22 had more strong selection on the production performance than S21 pigs (34), and S21 may have a special selection for reproduction and S22 may have a special selection for immunity (43). In the breeding program, superior individuals always had more mating opportunities than inferior individuals, which may reduce the number of harmful ROHs in the population. In consistent with previous literature (23, 44), reproductive trait (TTN in this study) generally had a lower number of significantly adverse ROHs than that of other four productive traits. This may be due to reproductive traits have a relatively lower heritability and are largely affected by environmental conditions and management strategies (45). The unfavorable ROHs were randomly distributed on the genome. In general, S22 had larger average, minimum, and maximum (except for ADG) ROH lengths than S21 pigs. This revealed that harmful genotypes were more accumulated in recent generations in S22, which may be caused by the stronger selection. According to the formula *L*_*ROH*_ = 100/(2 g^*^cM), *L*_*ROH*_ is the length of ROH, g is the generation age, and 1 cM ≈ 1 Mb (46). The maximum length of significantly adverse ROHs was 12.43 Mb on the LMA in S21 pigs and 9.02 Mb on the BFT in S22 pigs, implying that these two ROHs were formed more than 4.02 and 5.54 generations ago, respectively.

We mainly focused on the potential candidate genes in the overlapping ROHs with significantly adverse effects on multiple economic traits. Because these common ROH genotypes negatively related to multiple traits were sensitive to inbreeding and thus strongly reduced the overall performance of individual with these segments (21, 22). We observed that ADG had more completely or partially shared ROHs with other traits in S21 pigs, and BFT had five overlapping ROHs with LMP in S22 pigs. This may be caused by genetic correlations between traits, such as ADG had significant positive correlations with BFT, LMA, and LMP, and BFT had a significant negative correlation with LMP. As expected, a series of genes involved in fat deposition, growth, skeletal, and muscle development were found in these pleiotropic ROHs. In addition, a list of genes related to immunity, spermatogenesis, and embryonic development were detected in pleiotropic ROH regions in two Duroc pigs. It is easy to understand that immunity is directly related to health and production performance in an individual. A previous literature (47) reported that abnormalities in gametogenesis, embryonic development, and intrauterine environment may cause health problems for offspring, which may present as birth defects, growth retardation, and other chronic metabolic diseases.

We highlighted the overlapping ROH regions with unfavorable effects on three (ADG, LMA, and LMP; SSC18:38.51–40.79 Mb) and four traits (BFT, LMA, LMP, and TTN; SSC18:43.60–44.00 Mb) in S21 pigs. A total of three functional genes were located on three-trait pleiotropic ROH, such as *DPY19L2* gene is related to spermatogenesis and embryonic development (48); *BMPER* null mutants lead to prenatal lethality with skeletal malformations in both human and mice (49, 50); Loss-of-function mutations in human BSS9 cause Bardet–Biedl syndrome, including obesity, renal anomalies, and retinopathy (51). A 212-kb deletion within the *BBS9* gene has antagonistic effects on fertility and growth in pigs (52). The homozygous state reduces expression of the downstream *BMPER* gene resulting in fetal death. In contrast, the heterozygous state shows a positive effect on growth rate and feed intake. Therefore, maintaining the polymorphism of this unfavorable ROH region may show a heterozygote advantage for growth rate, which is an important target of commercial pig breeding. There were two functional genes located on four-trait pleiotropic ROH, and *CPVL* has a biased expression in ovary and is associated with the digestive breakdown of proteins in the gut and immunity (53); *CREB5* gene is involved in adipocyte differentiation (54).

Moreover, two overlapping ROHs with significantly adverse effects on ADG and BFT traits were identified in two Duroc lines. ROH segment associated with ADG was located on SSC7 (20.05–20.30 Mb), containing the *CARMIL1* gene. *CARMIL1* was a candidate gene associated with ACTH concentration, which was positively correlated with body weight, cannon circumference, and hip width in cattle (55). We also detected a significant harmful ROH (SSC18:44.82–46.52 Mb) associated with BFT in two Duroc populations, harboring nine lipid accumulation-related genes including *JAZF1* and eight homeobox A genes (e.g., *HOXA1, HOXA2, HOXA3, HOXA5, HOXA7, HOXA10, HOXA11*, and *HOXA13*). *JAZF1* plays an important role in regulating lipid homeostasis (56). Numerous studies reported that these homeobox A genes were involved in adipogenesis and lipid metabolism (57–60). Previous research (61) using ROH and selective sweep analyses revealed that Chinese Jinhua pigs had a positive selection in the region of SSC18:45.20–46.25 Mb. *HOXA3, HOXA7, HOXA10*, and *HOXA11* genes were reported to affect embryo implantation and prolificacy traits (62–64). Jinhua pig is a well-known native breed in eastern China that has excellent fertility. This genomic region may be selected to improve the reproduction performance of Jinhua pigs. Obesity is closely related to reproduction and fertility, such as embryonic development (65) and spermatogenesis (66) through various mechanisms. Low BFT is an important breeding objective for Duroc pigs, and animals carrying this adverse ROH showed higher BFT values than Non-carriers. We hypothesize that the homozygosity of this genomic region increased the BFT in Duroc pigs, but it may improve the poor female reproductive performance of Duroc pigs.

We observed that the average frequencies of significantly unfavorable ROH were low (5.80 and 6.82%) in two Duroc pigs. This is possibly because these two populations were bred in modern commercial farms with excellent feeding and inbreeding control managements, which may reduce the accumulation of adverse ROHs in two populations to some extent. The results were similar to previous study (67) that specific breeding programs can counterbalance the effects of inbreeding in commercial chicken lines. In addition, commercial lines performed the phenotypic selection in each generation and discarded individuals with lower levels of phenotypic performances, which may carry more unfavorable ROHs. Our results also showed that unfavorable ROHs were diversely dispersed in two Duroc line genomes, complicating strategies to eradicate these harmful haplotypes *via* individual selection. Currently, commercial pig breeding has entered the era of genomic selection, which has greatly increased the production performances. However, genomic selection focuses more on stacking beneficial genes and alleles, while ignoring the influence of unfavorable loci on phenotype. Hence, we suggest that the information of unfavorable ROHs can, and should, be included and utilized in the breeding programs, which may play an assisting role in the genetic improvement of pigs. Previous studies (21–23) have suggested that identifying and discarding individuals with these adverse genotypes in mating programs can minimize the frequency of the harmful ROHs in the population and would be beneficial to avoid the unfavorable effect of inbreeding depression. However, our results revealed that some ROH regions may play opposite roles in different traits. Therefore, the effects of ROH on different phenotypes should be correctly evaluated and weighted in future breeding programs. Moreover, artificial selection may simultaneously enhance the homozygosity of beneficial and deleterious mutations, and inbreeding depression is sometimes caused by major effects at a few detrimental loci (68). Therefore, effective control of inbreeding depression requires identifying harmful alleles and understanding their effects on different phenotypes, rather than simply avoiding the mating of any ROH carriers (22). Due to the limitation of the chip density used in this study, we did not detect the casual deleterious mutations. Further, in-depth studies, such as whole-genome resequencing, RNA-sequencing, and functional experiments, can validate and improve our results.

## Conclusion

In summary, this work investigated the association between ROH and five economic traits in two Duroc pig lines. The results revealed that the number of unfavorable ROH genotypes had significant difference between two Duroc lines, which may be related to the different selection directions and intensities in two populations. We observed that many pleiotropic ROHs had consistent adverse effects across multiple traits, and candidate genes were mainly related to growth, fertility, and immunity. Hence, these harmful ROH segments can be used as the indicators to detect and manage inbreeding depression in populations. We highlighted that several unfavorable ROHs possibly had opposite effects in different traits. Therefore, correctly understanding and balancing the roles of unfavorable ROHs among different phenotypes may provide new insights into the genetic improvement of pigs.

## Data Availability Statement

The datasets presented in this study can be found in online repositories. The names of the repository/repositories and accession number(s) can be found below: https://doi.org/10.6084/m9.figshare.8019551.v1.

## Ethics Statement

The animal study was reviewed and approved by Animal Care and Use Committee of the South China Agricultural University.

## Author Contributions

ZW, JY, and XW: proposed the idea of study and directed the analyses and revised the manuscript. SW: performed the analysis and wrote the manuscript. GL, RD, ZZ, DR, JW, HY, EZ, and GC: collected samples and performed the experiments. ZW and JY: contributed the materials. All authors reviewed and approved the manuscript. All authors contributed to the article and approved the submitted version.

## Funding

This research was funded by the Project of Swine Innovation Team in Guangdong Modern Agricultural Research System (2021KJ126), the Local Innovative and Research Teams Project of Guangdong Province (2019BT02N630), and the National High-quality lean-type Pig Breeding United Research Program of China.

## Conflict of Interest

The authors declare that the research was conducted in the absence of any commercial or financial relationships that could be construed as a potential conflict of interest.

## Publisher's Note

All claims expressed in this article are solely those of the authors and do not necessarily represent those of their affiliated organizations, or those of the publisher, the editors and the reviewers. Any product that may be evaluated in this article, or claim that may be made by its manufacturer, is not guaranteed or endorsed by the publisher.

## Supplementary Material

Supplementary Figure S1 Correlation analyses among ROH and five economic traits in two Duroc populations.

Supplementary Table S1 Information on five economic traits of two Duroc pig lines.

Supplementary Table S2 Significantly unfavorable ROHs on five economic traits in S21 pigs.

Supplementary Table S3 Significantly unfavorable ROHs on four economic traits in S22 pigs.

Supplementary Table S4 Function of candidate genes in the significantly unfavorable ROHs.
