# Supplementary material for: Identification of Homozygous Regions With Adverse Effects on the Five Economic Traits of Duroc Pigs
Source: Front Vet Sci. 2022 Apr 28;9:855933. doi: 10.3389/fvets.2022.855933 (PMC9096619; doi:10.3389/fvets.2022.855933)
Supplement: Supplementary Figure S1 — Correlation analyses among ROH and five economic traits in two Duroc populations. [file Data_Sheet_1.docx]

**Supplementary files**


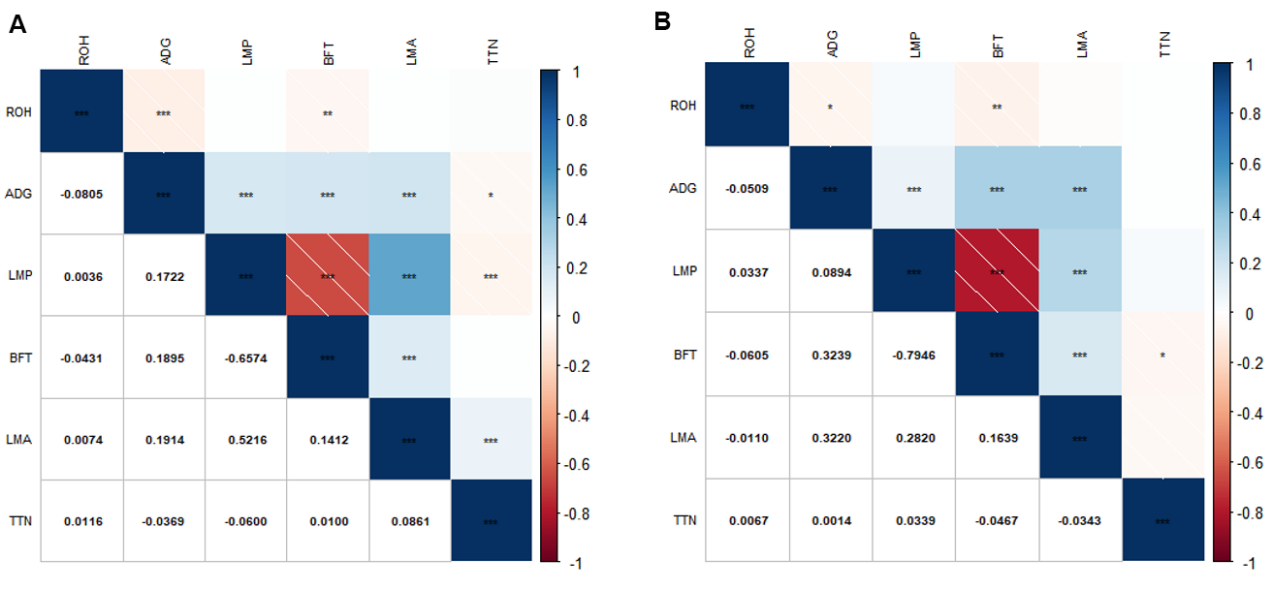


Supplementary Figure S1: Correlation analyses among ROH and five economic traits in two Duroc populations. (A) S21 pigs. (B) S22 pigs. S21, Duroc pigs of American origin; S22, Duroc pigs of Canadian origin; ROH, total ROH length of each individual; ADG, average daily gain at 100 kg; BFT, backfat thickness at 100 kg; LMA, loin muscle area at 100 kg; LMP, lean meat percentage at 100 kg; and TTN, total teat number.

Supplementary Table S1. Information on five economic traits of two Duroc pig lines.

| **Breed** | **Traits** | **Fixed effects** | **Genetic parameter** | | **Animals (records)** | | |
| --- | --- | --- | --- | --- | --- | --- | --- |
|  |  |  | **h^2^** | **SE** | **No** | **Mean** | **SD** |
| S21 | ADG (g) | Sex, Years | 0.26 | 0.02 | 3770 | 619.4 | 32.70 |
|  | BFT (mm) | Sex, Years, Days | 0.34 | 0.02 | 3770 | 10.30 | 1.32 |
|  | LMA (cm2) | Sex, Years, Days | 0.37 | 0.02 | 3770 | 42.31 | 3.85 |
|  | LMP (%) | Sex, Years, Weight | 0.30 | 0.02 | 3770 | 62.24 | 1.00 |
|  | TTN | Sex, Years | 0.19 | 0.02 | 3770 | 11.34 | 1.09 |
| S22 | ADG (g) | Sex, Years | 0.28 | 0.03 | 2096 | 613.75 | 43.19 |
|  | BFT (mm) | Sex, Years, Days | 0.38 | 0.03 | 2096 | 11.10 | 2.33 |
|  | LMA (cm2) | Sex, Years, Days | 0.37 | 0.03 | 2096 | 39.07 | 4.35 |
|  | LMP (%) | Sex, Years, Weight | 0.36 | 0.03 | 2096 | 61.03 | 1.51 |
|  | TTN | Sex, Years | 0.34 | 0.03 | 2096 | 10.91 | 1.13 |

S21, Duroc pigs of American origin; S22, Duroc pigs of Canadian origin; Fixed effects, the fixed effects in linear mixed model for each trait; ADG, average daily gain at 100 kg; BFT, backfat thickness at 100 kg; LMA, loin muscle area at 100 kg; LMP, lean meat percentage at 100 kg; and TTN, total teat number; h^2^ (SE), heritability (standard error); No, number of records; Mean (SD), average of phenotypic values (standard deviation).

Supplementary Table S2. Significantly unfavorable ROHs on five economic traits in S21 pigs.

| **Chromosome** | **StartPos** | **EndPos** | **SNP** | **ROH Effect** | **T-Stat** | **-log10(p)** | **Frequency (%)** | **Trait** |
| --- | --- | --- | --- | --- | --- | --- | --- | --- |
| 1 | 7423138 | 9601235 | 54 | -1.19 | -3.39 | 3.46 | 2.97 | LMA |
| 1 | 251406794 | 253265267 | 50 | 0.35 | 3.98 | 4.45 | 9.50 | BFT |
| 1 | 251781867 | 253822166 | 50 | 0.36 | 4.23 | 4.92 | 9.84 | BFT |
| 2 | 7960262 | 9614500 | 50 | -1.37 | -3.09 | 3.00 | 1.83 | LMA |
| 2 | 11711274 | 13143791 | 51 | -1.29 | -2.94 | 2.78 | 1.88 | LMA |
| 2 | 13970458 | 16448563 | 50 | -0.75 | -3.13 | 3.06 | 6.66 | LMA |
| 2 | 120581732 | 122988585 | 50 | -1.75 | -3.09 | 3.00 | 1.11 | LMA |
| 2 | 132946181 | 134669139 | 50 | -1.60 | -4.20 | 4.87 | 2.52 | LMA |
| 2 | 138424173 | 139741258 | 54 | -16.59 | -2.94 | 2.79 | 0.77 | ADG |
| 3 | 23621975 | 26196960 | 52 | -0.22 | -3.29 | 3.29 | 5.84 | LMP |
| 3 | 25852288 | 28715680 | 52 | -0.22 | -3.20 | 3.16 | 5.12 | LMP |
| 3 | 70206783 | 73204493 | 50 | -6.15 | -3.26 | 3.25 | 7.37 | ADG |
| 3 | 71835301 | 75252723 | 51 | -6.29 | -3.19 | 3.15 | 6.68 | ADG |
| 3 | 72566684 | 75679556 | 51 | -0.98 | -3.69 | 3.94 | 5.33 | LMA |
| 3 | 103709780 | 106282920 | 54 | -0.38 | -3.12 | 3.04 | 1.59 | LMP |
| 3 | 112161506 | 113981918 | 50 | -0.36 | -3.50 | 3.64 | 2.25 | LMP |
| 4 | 7234043 | 8302925 | 51 | -1.01 | -2.98 | 2.84 | 3.16 | LMA |
| 4 | 7305995 | 8403678 | 51 | -1.07 | -3.13 | 3.06 | 3.10 | LMA |
| 4 | 7472267 | 8649410 | 50 | -1.09 | -3.29 | 3.29 | 3.34 | LMA |
| 4 | 7965830 | 9354508 | 50 | -1.09 | -3.16 | 3.10 | 3.08 | LMA |
| 4 | 76574796 | 80408198 | 52 | 0.72 | 3.15 | 3.08 | 1.27 | BFT |
| 5 | 3367985 | 4636641 | 50 | -9.72 | -3.51 | 3.64 | 3.26 | ADG |
| 5 | 9683874 | 10879898 | 50 | -9.35 | -2.95 | 2.79 | 2.47 | ADG |
| 5 | 24085936 | 27908861 | 51 | -0.98 | -3.78 | 4.09 | 5.65 | LMA |
| 5 | 45935649 | 54059989 | 51 | -0.69 | -4.61 | 5.68 | 20.00 | LMA |
| 5 | 46266348 | 54923971 | 50 | -0.65 | -4.29 | 5.03 | 19.34 | LMA |
| 5 | 50860053 | 57631586 | 50 | -0.66 | -4.13 | 4.73 | 17.14 | LMA |
| 5 | 66527456 | 69449751 | 50 | -0.99 | -3.27 | 3.27 | 4.06 | LMA |
| 6 | 46159717 | 49643149 | 53 | -6.94 | -3.61 | 3.80 | 7.11 | ADG |
| 6 | 68831062 | 71877417 | 51 | -0.63 | -3.65 | 3.88 | 14.08 | LMA |
| 6 | 68831062 | 71877417 | 51 | -0.18 | -4.02 | 4.53 | 14.08 | LMP |
| 6 | 69366409 | 72378637 | 52 | -0.22 | -3.17 | 3.12 | 5.36 | LMP |
| 6 | 130937009 | 134840192 | 51 | -0.68 | -3.99 | 4.47 | 14.03 | LMA |
| 6 | 131175703 | 134842583 | 50 | -0.67 | -3.93 | 4.36 | 14.08 | LMA |
| 6 | 131366300 | 135297800 | 53 | -0.70 | -4.07 | 4.63 | 14.08 | LMA |
| 6 | 131595818 | 135347154 | 50 | -0.69 | -4.00 | 4.50 | 14.11 | LMA |
| 7 | 20052479 | 23037876 | 51 | -5.56 | -3.18 | 3.12 | 8.73 | ADG |
| 7 | 40892446 | 43209269 | 50 | -0.27 | -3.87 | 4.27 | 5.23 | LMP |
| 7 | 40992620 | 43225630 | 50 | -0.27 | -3.89 | 4.30 | 5.23 | LMP |
| 7 | 41001684 | 43245762 | 50 | -0.28 | -4.09 | 4.65 | 5.41 | LMP |
| 7 | 99269726 | 101808111 | 50 | -0.24 | -3.42 | 3.49 | 5.15 | LMP |
| 7 | 99716434 | 102170676 | 50 | -0.22 | -3.16 | 3.09 | 5.31 | LMP |
| 8 | 7107413 | 8409222 | 51 | -0.41 | -3.46 | 3.56 | 2.33 | TTN |
| 8 | 7154884 | 8423305 | 50 | -0.42 | -3.58 | 3.77 | 2.41 | TTN |
| 8 | 24980021 | 27902238 | 51 | -1.23 | -3.21 | 3.18 | 2.47 | LMA |
| 8 | 40025212 | 52458798 | 54 | -0.75 | -3.01 | 2.89 | 6.05 | LMA |
| 8 | 83078635 | 86582725 | 50 | -0.61 | -3.12 | 3.04 | 10.40 | LMA |
| 8 | 85010670 | 87508941 | 51 | -0.60 | -3.10 | 3.01 | 10.53 | LMA |
| 8 | 85159743 | 87509059 | 50 | -0.62 | -3.20 | 3.16 | 10.61 | LMA |
| 8 | 94481196 | 99298999 | 50 | -9.16 | -3.83 | 4.18 | 4.51 | ADG |
| 9 | 50294687 | 53715696 | 50 | -1.00 | -3.10 | 3.01 | 3.53 | LMA |
| 9 | 55113186 | 58018443 | 52 | -1.33 | -2.92 | 2.76 | 1.75 | LMA |
| 9 | 73292979 | 82558733 | 50 | -9.25 | -3.50 | 3.63 | 3.63 | ADG |
| 9 | 128084622 | 129385280 | 53 | -2.24 | -3.23 | 3.21 | 0.74 | LMA |
| 10 | 27537037 | 32225456 | 54 | -15.60 | -3.16 | 3.10 | 1.01 | ADG |
| 12 | 11867363 | 14993375 | 53 | -0.47 | -3.72 | 3.99 | 1.46 | LMP |
| 12 | 19805312 | 22286305 | 50 | -0.77 | -3.08 | 2.98 | 6.10 | LMA |
| 12 | 44491255 | 47083296 | 51 | -1.04 | -2.98 | 2.84 | 3.00 | LMA |
| 12 | 50398492 | 53273240 | 53 | -12.10 | -3.02 | 2.90 | 1.54 | ADG |
| 13 | 8191206 | 9230860 | 51 | -1.07 | -3.08 | 2.98 | 3.02 | LMA |
| 13 | 8234275 | 9332186 | 51 | -1.04 | -3.03 | 2.91 | 3.10 | LMA |
| 13 | 25755813 | 27441401 | 50 | -0.23 | -3.70 | 3.95 | 8.75 | TTN |
| 13 | 130751480 | 135435438 | 51 | -0.94 | -3.46 | 3.56 | 5.07 | LMA |
| 14 | 30662019 | 33711965 | 51 | -6.74 | -3.86 | 4.23 | 8.89 | ADG |
| 14 | 101650509 | 104854343 | 53 | -0.58 | -3.60 | 3.80 | 16.79 | LMA |
| 14 | 102286461 | 105428138 | 52 | -0.62 | -4.03 | 4.55 | 18.41 | LMA |
| 14 | 102414475 | 105443888 | 50 | -0.62 | -4.03 | 4.54 | 18.46 | LMA |
| 14 | 137463463 | 139717828 | 50 | -9.44 | -2.95 | 2.80 | 2.44 | ADG |
| 14 | 138542277 | 141410475 | 52 | -0.30 | -3.60 | 3.79 | 4.85 | TTN |
| 15 | 3204205 | 4781569 | 50 | -0.70 | -3.59 | 3.78 | 10.56 | LMA |
| 15 | 3258523 | 4841509 | 51 | -0.70 | -3.59 | 3.78 | 10.58 | LMA |
| 15 | 3332599 | 4877567 | 50 | -0.70 | -3.59 | 3.78 | 10.61 | LMA |
| 15 | 3401591 | 4960376 | 50 | -0.70 | -3.56 | 3.72 | 10.29 | LMA |
| 15 | 7656967 | 9745391 | 51 | -1.62 | -3.15 | 3.08 | 1.35 | LMA |
| 15 | 37230998 | 42528646 | 50 | -4.83 | -2.94 | 2.79 | 10.08 | ADG |
| 15 | 95715165 | 101084148 | 52 | -11.83 | -2.98 | 2.84 | 1.56 | ADG |
| 15 | 103162393 | 108848114 | 54 | -7.59 | -3.17 | 3.11 | 4.46 | ADG |
| 15 | 103162393 | 108848114 | 54 | -1.14 | -3.93 | 4.36 | 4.46 | LMA |
| 15 | 111983184 | 115091121 | 52 | -1.43 | -3.65 | 3.88 | 2.36 | LMA |
| 15 | 116438713 | 119060372 | 54 | -1.17 | -3.00 | 2.86 | 2.36 | LMA |
| 15 | 119830414 | 121460992 | 52 | -1.11 | -3.58 | 3.75 | 3.79 | LMA |
| 15 | 120863537 | 122463289 | 50 | -1.10 | -3.52 | 3.65 | 3.79 | LMA |
| 15 | 120938602 | 122503503 | 51 | -1.12 | -3.67 | 3.90 | 3.95 | LMA |
| 15 | 121570230 | 122979514 | 50 | -1.26 | -4.22 | 4.90 | 4.16 | LMA |
| 15 | 122539331 | 123601682 | 51 | 0.31 | 3.15 | 3.08 | 7.40 | BFT |
| 15 | 130356053 | 132308830 | 52 | -0.51 | -2.92 | 2.76 | 13.66 | LMA |
| 15 | 130946387 | 132589115 | 50 | -0.16 | -3.71 | 3.98 | 24.24 | TTN |
| 15 | 136149781 | 137874383 | 51 | -0.25 | -3.59 | 3.77 | 5.09 | LMP |
| 15 | 136310724 | 138310780 | 53 | -0.24 | -3.10 | 3.01 | 4.16 | LMP |
| 16 | 605799 | 2406362 | 52 | -8.95 | -3.26 | 3.25 | 3.34 | ADG |
| 16 | 16650413 | 19015840 | 51 | -5.96 | -3.32 | 3.35 | 8.28 | ADG |
| 16 | 17757484 | 19941695 | 50 | -5.94 | -3.34 | 3.37 | 8.44 | ADG |
| 16 | 26261784 | 29292557 | 51 | -4.95 | -3.97 | 4.44 | 19.79 | ADG |
| 16 | 27280636 | 30283095 | 50 | -4.97 | -4.12 | 4.72 | 21.59 | ADG |
| 16 | 27314294 | 30557689 | 51 | -5.01 | -4.16 | 4.79 | 21.67 | ADG |
| 16 | 27463044 | 30605251 | 50 | -4.98 | -4.13 | 4.74 | 21.64 | ADG |
| 16 | 27623105 | 31102868 | 53 | -0.74 | -4.88 | 6.26 | 19.44 | LMA |
| 16 | 52092180 | 55142869 | 51 | -7.96 | -3.01 | 2.88 | 3.61 | ADG |
| 16 | 52740432 | 55257771 | 50 | -1.03 | -3.32 | 3.34 | 3.82 | LMA |
| 16 | 52981933 | 55655094 | 54 | -8.31 | -3.22 | 3.19 | 3.79 | ADG |
| 16 | 53580753 | 55871009 | 51 | -1.02 | -3.35 | 3.40 | 3.98 | LMA |
| 16 | 55940212 | 59008528 | 50 | -1.20 | -3.14 | 3.07 | 2.47 | LMA |
| 16 | 67360520 | 69411917 | 50 | -0.24 | -3.38 | 3.43 | 4.80 | LMP |
| 17 | 15659761 | 17867190 | 52 | -11.24 | -3.79 | 4.12 | 2.84 | ADG |
| 17 | 27809750 | 30088112 | 51 | -1.87 | -2.89 | 2.72 | 0.85 | LMA |
| 18 | 20535801 | 23493145 | 51 | -0.50 | -4.14 | 4.74 | 1.64 | LMP |
| 18 | 37609289 | 40790150 | 50 | -0.22 | -3.68 | 3.92 | 7.43 | LMP |
| 18 | 37883664 | 41007612 | 50 | -6.84 | -3.44 | 3.52 | 6.58 | ADG |
| 18 | 38024213 | 41089501 | 51 | -6.69 | -3.37 | 3.41 | 6.60 | ADG |
| 18 | 38506661 | 41269203 | 50 | -0.82 | -3.19 | 3.14 | 5.73 | LMA |
| 18 | 42050154 | 44002242 | 50 | -1.75 | -4.17 | 4.80 | 2.07 | LMA |
| 18 | 43459271 | 46254928 | 50 | -0.27 | -3.38 | 3.44 | 5.31 | TTN |
| 18 | 43597777 | 46523411 | 52 | 0.82 | 3.23 | 3.20 | 1.03 | BFT |
| 18 | 43597777 | 46523411 | 52 | -0.55 | -3.61 | 3.82 | 1.03 | LMP |

Supplementary Table S3 Significantly unfavorable ROHs on four economic traits in S22 pigs.

| **Chromosome** | **StartPos** | **EndPos** | **SNP** | **ROH Effect** | **T-Stat** | **-log10(p)** | **Frequency (%)** | **Trait** |
| --- | --- | --- | --- | --- | --- | --- | --- | --- |
| 1 | 53753531 | 59133134 | 51 | -0.49 | -2.90 | 2.72 | 4.15 | LMP |
| 1 | 86116155 | 94245906 | 55 | -19.45 | -3.45 | 3.54 | 2.67 | ADG |
| 1 | 229487778 | 236472874 | 50 | -0.79 | -3.48 | 3.60 | 2.24 | LMP |
| 1 | 229638669 | 236815238 | 52 | -0.74 | -3.35 | 3.39 | 2.34 | LMP |
| 1 | 248654503 | 251015010 | 50 | -0.42 | -3.86 | 4.23 | 10.54 | LMP |
| 1 | 248999553 | 251406794 | 50 | -0.44 | -4.07 | 4.61 | 10.69 | LMP |
| 1 | 257435300 | 259260476 | 50 | 2.72 | 5.79 | 8.38 | 1.24 | BFT |
| 1 | 257435300 | 259260476 | 50 | -1.47 | -4.88 | 6.24 | 1.24 | LMP |
| 1 | 257591346 | 259559779 | 51 | 0.78 | 3.06 | 2.96 | 4.39 | BFT |
| 2 | 5983861 | 7705804 | 50 | 1.20 | 2.91 | 2.74 | 1.62 | BFT |
| 2 | 7122345 | 8894947 | 51 | 1.48 | 3.53 | 3.67 | 1.57 | BFT |
| 2 | 31158784 | 36448775 | 53 | -15.48 | -3.15 | 3.08 | 3.48 | ADG |
| 2 | 116674257 | 118756751 | 50 | 1.02 | 2.94 | 2.78 | 2.29 | BFT |
| 2 | 121253653 | 123679815 | 50 | -0.43 | -3.05 | 2.93 | 5.96 | LMP |
| 2 | 135687321 | 137475641 | 53 | 1.54 | 4.26 | 4.97 | 2.15 | BFT |
| 2 | 135687321 | 137475641 | 53 | -0.91 | -3.94 | 4.38 | 2.15 | LMP |
| 3 | 7826355 | 10104448 | 50 | 0.53 | 3.48 | 3.60 | 13.65 | BFT |
| 3 | 108958589 | 110779313 | 52 | 0.65 | 3.19 | 3.14 | 7.11 | BFT |
| 3 | 128568743 | 130918532 | 51 | 0.89 | 4.59 | 5.63 | 7.82 | BFT |
| 3 | 129057670 | 131834874 | 54 | 0.65 | 3.85 | 4.22 | 10.73 | BFT |
| 3 | 129057670 | 131834874 | 54 | -0.38 | -3.48 | 3.59 | 10.73 | LMP |
| 3 | 129412019 | 132001703 | 50 | -0.40 | -3.72 | 4.00 | 10.88 | LMP |
| 4 | 66094932 | 69748937 | 50 | 0.61 | 3.72 | 3.99 | 11.50 | BFT |
| 4 | 66094932 | 69748937 | 50 | -0.44 | -4.15 | 4.76 | 11.50 | LMP |
| 4 | 66503208 | 70563762 | 50 | 0.73 | 4.37 | 5.18 | 10.93 | BFT |
| 4 | 91403268 | 93744042 | 53 | -10.25 | -3.33 | 3.36 | 9.73 | ADG |
| 4 | 91637982 | 94024286 | 51 | -10.20 | -3.52 | 3.66 | 11.12 | ADG |
| 5 | 39503733 | 47424047 | 54 | -13.90 | -3.09 | 3.00 | 4.29 | ADG |
| 6 | 128148550 | 131692557 | 50 | -11.85 | -3.14 | 3.07 | 6.20 | ADG |
| 7 | 13207826 | 15512745 | 50 | -9.10 | -3.47 | 3.58 | 14.07 | ADG |
| 7 | 17112442 | 20299952 | 50 | -10.87 | -3.28 | 3.27 | 8.21 | ADG |
| 7 | 95385191 | 98483627 | 50 | -0.60 | -4.04 | 4.56 | 3.15 | TTN |
| 8 | 86475921 | 88296664 | 50 | -0.33 | -2.97 | 2.83 | 10.59 | LMP |
| 8 | 118968215 | 120559374 | 51 | 1.17 | 2.95 | 2.79 | 1.77 | BFT |
| 8 | 119044777 | 120600278 | 50 | 1.29 | 3.29 | 3.29 | 1.81 | BFT |
| 9 | 38855733 | 40924173 | 50 | -0.88 | -3.18 | 3.13 | 1.48 | LMP |
| 9 | 65280510 | 74303991 | 50 | 0.72 | 2.89 | 2.71 | 4.53 | BFT |
| 10 | 31523619 | 35589467 | 52 | -0.39 | -3.44 | 3.53 | 5.58 | TTN |
| 10 | 45063224 | 48579723 | 50 | -0.95 | -3.30 | 3.31 | 1.38 | LMP |
| 10 | 54761683 | 57325327 | 50 | -0.73 | -3.11 | 3.03 | 2.10 | LMP |
| 12 | 5426010 | 7718201 | 50 | 1.39 | 3.16 | 3.10 | 1.43 | BFT |
| 12 | 18848981 | 21643703 | 51 | -15.32 | -3.08 | 2.98 | 3.44 | ADG |
| 12 | 19155862 | 21842539 | 51 | -16.12 | -3.26 | 3.24 | 3.48 | ADG |
| 12 | 19210822 | 21858483 | 51 | -15.69 | -3.25 | 3.24 | 3.67 | ADG |
| 13 | 13330289 | 15461954 | 50 | 0.72 | 4.16 | 4.79 | 10.31 | BFT |
| 13 | 13346860 | 15557104 | 50 | 0.73 | 4.22 | 4.90 | 10.35 | BFT |
| 13 | 13367835 | 15708162 | 50 | 0.69 | 4.08 | 4.64 | 10.59 | BFT |
| 13 | 18330154 | 21124020 | 54 | 1.59 | 3.11 | 3.02 | 1.05 | BFT |
| 13 | 19797590 | 21969196 | 52 | -0.35 | -3.20 | 3.15 | 10.50 | LMP |
| 13 | 42557867 | 47263536 | 54 | 0.61 | 3.44 | 3.52 | 9.73 | BFT |
| 14 | 35165807 | 38601904 | 50 | -16.35 | -3.30 | 3.30 | 3.48 | ADG |
| 14 | 138357861 | 141309602 | 54 | 0.56 | 3.19 | 3.15 | 9.64 | BFT |
| 16 | 7565523 | 10894800 | 54 | -19.19 | -3.13 | 3.05 | 2.24 | ADG |
| 16 | 20264243 | 21884754 | 50 | -24.35 | -3.34 | 3.37 | 1.57 | ADG |
| 17 | 10123576 | 12835237 | 53 | -0.54 | -3.08 | 2.98 | 3.77 | LMP |
| 18 | 31089645 | 34311344 | 52 | -38.79 | -4.64 | 5.74 | 1.19 | ADG |
| 18 | 44824396 | 47268729 | 50 | 0.73 | 3.40 | 3.46 | 6.35 | BFT |
| 18 | 50606439 | 53234165 | 52 | 0.89 | 3.34 | 3.37 | 3.91 | BFT |

Supplementary Table S4 Function of candidate genes in the significantly unfavorable ROHs.

| Gene | Chr | Position (Mb) | Line | Economic trait | Function | Reference |
| --- | --- | --- | --- | --- | --- | --- |
| *ANTXR1* | 3 | 73.16-73.40 | S21 | ADG, LMA | Skeletal development | (1) |
| *GKN1* | 3 | 3:73.42-73.43 | S21 | ADG, LMA | Growth;  skeletal development;  fat deposition | (2,3) |
| *PPP3R1* | 3 | 72.77-72.83 | S21 | ADG, LMA | immune response | (4) |
| *PNO1* | 3 | 74.07-74.08 | S21 | ADG, LMA | immune response | (5) |
| *TARDBP* | 6 | 71.21-71.23 | S21 | LMA, LMP | fat deposition | (6) |
| *MTOR* | 6 | 71.29-71.41 | S21 | LMA, LMP | embryonic development | (7) |
| *SLC2A5* | 6 | 69.51-69.54 | S21 | LMA, LMP? | nutrient absorption | (8) |
| *H6PD* | 6 | 69.67-69.71 | S21 | LMA, LMP? | muscle development | (9) |
| *CASP8* | 15 | 104.92-104.95 | S21 | ADG, LMA | immune response | (10) |
| *BMPR2* | 15 | 105.97-106.14 | S21 | ADG, LMA | Embryonic development | (11) |
| *ICA1L* | 15 | 106.30-106.38 | S21 | ADG, LMA | gametogenesis | (12) |
| *ABI2* | 15 | 106.78-106.90 | S21 | ADG, LMA | immune response | (13) |
| *CD28* | 15 | 107.13-107.16 | S21 | ADG, LMA | immune response | (14) |
| *CTLA4* | 15 | 107.28-107.29 | S21 | ADG, LMA | immune response | (15) |
| *ICOS* | 15 | 107.35-107.37 | S21 | ADG, LMA | immune response | (16) |
| *GPR55* | 15 | 131.66-131.67 | S21 | LMA, TTN | Immune response;  obesity | (17) |
| *FGF10* | 16 | 28.69-28.78 | S21 | ADG, LMA | Growth;  skeletal development | (18) |
| *FGF18* | 16 | 52.73-52.76 | S21 | ADG, LMA | Growth;  skeletal development | (19) |
| *DOCK2* | 16 | 53.84-54.25 | S21 | ADG, LMA | Immune response | (20) |
| *DPY19L2* | 18 | 38.69-38.77 | S21 | ADG, LMA, LMP | Spermatogenesis;  embryonic development | (21) |
| *BMPER* | *18* | 39.41-39.95 | S21 | ADG, LMA, LMP | prenatal lethality with skeletal malformations | (22) |
| *BBS9* | 18 | 39.68-40.39 | S21 | ADG, LMA, LMP | Fertility;  Growth | (23) |
| *CPVL* | 18 | 43.56-43.69 | S21 | BFT, LMA, LMP, TTN | Fertility;  Immunity | (24) |
| *CREB5* | 18 | 43.84-44.26 | S21 | BFT, LMA, LMP, TTN | Adipocyte differentiation;  immunity | (25) |
| *TLR4* | 1 | 258.04-258.06 | S22 | BFT, LMP | obesity | (26) |
| *PPP2CA* | 2 | 136.49-136.52 | S22 | BFT, LMP | spermatogenesis | (27) |
| *UBE2B* | 2 | 136.65-136.67 | S22 | BFT, LMP | spermatogenesis | (28) |
| *CATSPER3* | 2 | 137.14-137.18 | S22 | BFT, LMP | spermatogenesis | (29) |
| *PITX1* | 2 | 137.20-137.21 | S22 | BFT, LMP | muscle development | (30) |
| *SOX11* | 3 | 129.79-129.80 | S22 | BFT, LMP | embryonic development | (31) |
| *CRH* | 4 | 68.48-68.49 | S22 | BFT, LMP | lipid metabolism | (32) |
| *CYP7B1* | 4 | 69.62-69.81 | S22 | BFT, LMP | fatty acid composition | (33) |
| *ARPP21* | 13 | 20.93-21.09 | S22 | BFT, LMP | muscle development | (34) |
| *CARMIL1* | 7 | 20.04-20.35 | S21, S22 | ADG | growth | (35) |
| *JAZF1* | 18 | 44.49-44.83 | S21, S22 | BFT | lipid accumulation | (36) |
| *HOXA1* | 18 | 45.47-45.48 | S21, S22 | BFT | lipid accumulation | (37) |
| *HOXA2* | 18 | 45.47-45.47 | S21, S22 | BFT | growth | (38) |
| *HOXA3* | 18 | 45.45-45.47 | S21, S22 | BFT | lipid accumulation | (39) |
| *HOXA5* | 18 | 45.42-45.43 | S21, S22 | BFT | lipid accumulation | (37) |
| *HOXA7* | 18 | 45.41-45.42 | S21, S22 | BFT | skeletal development | (40) |
| *HOXA10* | 18 | 45.39-45.40 | S21, S22 | BFT | lipid accumulation | (37) |
| *HOXA11* | 18 | 45.38-45.39 | S21, S22 | BFT | immunity | (41) |
| *HOXA13* | 18 | 45.37-45.38 | S21, S22 | BFT | skeletal development | (42) |

**References**

1. Jiang, Q., Qin, X., Yoshida, C.A., Komori, H., Yamana, K., Ohba, S., et al. (2020). Antxr1, which is a target of runx2, regulates chondrocyte proliferation and apoptosis. Int J Mol Sci. 21: 2425. doi:10.3390/ijms21072425

2. Overstreet, A.C., Grayson, B.E., Boger, A., Bakke, D., Carmody, E.M., Bales, C.E., et al. (2021). Gastrokine-1, an anti-amyloidogenic protein secreted by the stomach, regulates diet-induced obesity. Sci Rep. 11: 9477. doi:10.1038/s41598-021-88928-8

3. Yoon, J.H., Seo, H.S., Choi, W.S., Kim, O., Nam, S.W., Lee, J.Y., et al. (2014). Gastrokine 1 induces senescence and apoptosis through regulating telomere length in gastric cancer. Oncotarget. 5: 11695-11708. doi:10.18632/oncotarget.2586

4. Sun, Z., Xia, W., Lyu, Y., Song, Y., Wang, M., Zhang, R., et al. (2021). Immune-related gene expression signatures in colorectal cancer. Oncol Lett. 22: 543. doi:10.3892/ol.2021.12804

5. Wang, X., Wu, T., Hu, Y., Marcinkiewicz, M., Qi, S., Valderrama-Carvajal, H., et al. (2012). Pno1 tissue-specific expression and its functions related to the immune responses and proteasome activities. Plos One. 7: e46093. doi:10.1371/journal.pone.0046093

6. Chiang, P.M., Ling, J., Jeong, Y.H., Price, D.L., Aja, S.M., Wong, P.C. (2010). Deletion of TDP-43 down-regulates Tbc1d1, a gene linked to obesity, and alters body fat metabolism. Proc Natl Acad Sci U S A. 107: 16320-16324. doi:10.1073/pnas.1002176107

7. Hwang, M., Perez, C.A., Moretti, L., Lu, B. (2008). The mTOR signaling network: Insights from its role during embryonic development. Curr Med Chem. 15: 1192-1208. doi:10.2174/092986708784310459

8. Barone, S., Fussell, S.L., Singh, A.K., Lucas, F., Xu, J., Kim, C., et al. (2009). Slc2a5 (Glut5) is essential for the absorption of fructose in the intestine and generation of fructose-induced hypertension. J Biol Chem. 284: 5056-5066. doi:10.1074/jbc.M808128200

9. Bauckneht, M., Pastorino, F., Castellani, P., Cossu, V., Orengo, A.M., Piccioli, P., et al. (2020). Increased myocardial (18)F-FDG uptake as a marker of Doxorubicin-induced oxidative stress. J Nucl Cardiol. 27: 2183-2194. doi:10.1007/s12350-019-01618-x

10. Zou, J., Xia, H., Zhang, C., Xu, H., Tang, Q., Zhu, G., et al. (2021). Casp8 acts through A20 to inhibit PD-L1 expression: The mechanism and its implication in immunotherapy. Cancer Sci. 112: 2664-2678. doi:10.1111/cas.14932

11. Wang, J., Liu, W., Lu, W., Luo, X., Lin, Y., Liu, S., et al. (2022). Sodium tanshinone IIA sulfonate enhances the BMP9-BMPR2-Smad1/5/9 signaling pathway in rat pulmonary microvascular endothelial cells and human embryonic stem cell-derived endothelial cells. Biochem Pharmacol. 199: 114986. doi:10.1016/j.bcp.2022.114986

12. He, J., Xia, M., Tsang, W.H., Chow, K.L., Xia, J. (2015). ICA1L forms BAR-domain complexes with PICK1 and is crucial for acrosome formation in spermiogenesis. J Cell Sci. 128: 3822-3836. doi:10.1242/jcs.173534

13. Stanton, R.J., Prod'Homme, V., Purbhoo, M.A., Moore, M., Aicheler, R.J., Heinzmann, M., et al. (2014). HCMV pUL135 remodels the actin cytoskeleton to impair immune recognition of infected cells. Cell Host Microbe. 16: 201-214. doi:10.1016/j.chom.2014.07.005

14. Xia, S., Chen, Q., Niu, B. (2020). CD28: A new drug target for immune disease. Curr Drug Targets. 21: 589-598. doi:10.2174/1389450120666191114102830

15. Kuehn, H.S., Ouyang, W., Lo, B., Deenick, E.K., Niemela, J.E., Avery, D.T., et al. (2014). Immune dysregulation in human subjects with heterozygous germline mutations in CTLA4. Science. 345: 1623-1627. doi:10.1126/science.1255904

16. Xie, S., Wei, H., Peng, A., Xie, A., Li, J., Fang, C., et al. (2021). Ikzf2 regulates the development of ICOS^+^ th cells to mediate immune response in the spleen of s. Japonicum-Infected C57BL/6 mice. Front Immunol. 12: 687919. doi:10.3389/fimmu.2021.687919

17. Ayakannu, T., Taylor, A.H., Konje, J.C. (2021). Expression of the putative cannabinoid receptor GPR55 is increased in endometrial carcinoma. Histochem Cell Biol. 156: 449-460. doi:10.1007/s00418-021-02018-4

18. Lovinescu, I., Koyama, E., Pacifici, M. (2003). Roles of FGF-10 on the development of diathrodial limb joints. Penn Dent J. 103: 5, 9.

19. Xie, Y., Zinkle, A., Chen, L., Mohammadi, M. (2020). Fibroblast growth factor signalling in osteoarthritis and cartilage repair. Nat Rev Rheumatol. 16: 547-564. doi:10.1038/s41584-020-0469-2

20. Li, W., Li, J., He, N., Dai, X., Wang, Z., Wang, Y., et al. (2021). Molecular mechanism of enhancing the immune effect of the Newcastle disease virus vaccine in broilers fed with Bacillus cereus PAS38. Food Funct. 12: 10903-10916. doi:10.1039/d1fo01777b

21. Castaneda, J.M., Shimada, K., Satouh, Y., Yu, Z., Devlin, D.J., Ikawa, M., et al. (2021). FAM209 associates with DPY19L2, and is required for sperm acrosome biogenesis and fertility in mice. J Cell Sci. 134: jcs259206. doi:10.1242/jcs.259206

22. Greenbaum, L., Gilboa, Y., Raas-Rothschild, A., Barel, O., Kol, N., Reznik-Wolf, H., et al. (2019). Diaphanospondylodysostosis: Refining the prenatal diagnosis of a rare skeletal disorder. Eur J Med Genet. 62: 167-171. doi:10.1016/j.ejmg.2018.07.004

23. Sung, Y.J., Perusse, L., Sarzynski, M.A., Fornage, M., Sidney, S., Sternfeld, B., et al. (2016). Genome-wide association studies suggest sex-specific loci associated with abdominal and visceral fat. Int J Obes. 40: 662-674. doi:10.1038/ijo.2015.217

24. Yang, H., Liu, X., Zhu, X., Li, X., Jiang, L., Zhong, M., et al. (2021). CPVL promotes glioma progression via STAT1 pathway inhibition through interactions with the BTK/p300 axis. JCI Insight. 6: e146362. doi:10.1172/jci.insight.146362

25. Wang, S., Qiu, J., Liu, L., Su, C., Qi, L., Huang, C., et al. (2020). CREB5 promotes invasiveness and metastasis in colorectal cancer by directly activating MET. J Exp Clin Cancer Res. 39: 168. doi:10.1186/s13046-020-01673-0

26. Rogero, M.M., Calder, P.C. (2018). Obesity, Inflammation, Toll-Like Receptor 4 and Fatty Acids. Nutrients. 10: 432. doi:10.3390/nu10040432

27. Chen, X., Wang, W., Liu, X., Liu, H., Sun, H., Wang, L., et al. (2022). Catalytic subunit of protein phosphatase 2A (PP2Ac) influences the meiosis initiation during spermatocyte meiosis prophase i. Reprod Sci. doi:10.1007/s43032-022-00843-z

28. Yatsenko, A.N., Georgiadis, A.P., Murthy, L.J., Lamb, D.J., Matzuk, M.M. (2013). UBE2B mRNA alterations are associated with severe oligozoospermia in infertile men. Mol Hum Reprod. 19: 388-394. doi:10.1093/molehr/gat008

29. Wang, J., Tang, H., Zou, Q., Zheng, A., Li, H., Yang, S., et al. (2021). Patient with CATSPER3 mutations-related failure of sperm acrosome reaction with successful pregnancy outcome from intracytoplasmic sperm injection (ICSI). Mol Genet Genomic Med. 9: e1579. doi:10.1002/mgg3.1579

30. Wang, J.S., Infante, C.R., Park, S., Menke, D.B. (2018). PITX1 promotes chondrogenesis and myogenesis in mouse hindlimbs through conserved regulatory targets. Dev Biol. 434: 186-195. doi:10.1016/j.ydbio.2017.12.013

31. Miao, Q., Hill, M.C., Chen, F., Mo, Q., Ku, A.T., Ramos, C., et al. (2019). SOX11 and SOX4 drive the reactivation of an embryonic gene program during murine wound repair. Nat Commun. 10: 4042. doi:10.1038/s41467-019-11880-9

32. Wathes, D.C., Clempson, A.M., Pollott, G.E. (2012). Associations between lipid metabolism and fertility in the dairy cow. Reprod Fertil Dev. 25: 48-61. doi:10.1071/RD12272

33. Evangelakos, I., Schwinge, D., Worthmann, A., John, C., Roeder, N., Pertzborn, P., et al. (2021). Oxysterol 7-alpha hydroxylase (CYP7B1) attenuates Metabolic-Associated fatty liver disease in mice at thermoneutrality. Cells-Basel. 10: 2656. doi:10.3390/cells10102656

34. Tan, Y.Y., Zhang, Y., Li, B., Ou, Y.W., Xie, S.J., Chen, P.P., et al. (2021). PERK signaling controls myoblast differentiation by regulating MicroRNA networks. Front Cell Dev Biol. 9: 670435. doi:10.3389/fcell.2021.670435

35. Li, C., Stoma, S., Lotta, L.A., Warner, S., Albrecht, E., Allione, A., et al. (2020). Genome-wide association analysis in humans links nucleotide metabolism to leukocyte telomere length. Am J Hum Genet. 106: 389-404. doi:10.1016/j.ajhg.2020.02.006

36. Jeong, J., Jang, S., Park, S., Kwon, W., Kim, S.Y., Jang, S., et al. (2021). JAZF1 heterozygous knockout mice show altered adipose development and metabolism. Cell Biosci. 11: 161. doi:10.1186/s13578-021-00625-1

37. Singh, S., Rajput, Y.S., Barui, A.K., Sharma, R., Datta, T.K. (2016). Fat accumulation in differentiated brown adipocytes is linked with expression of Hox genes. Gene Expr Patterns. 20: 99-105. doi:10.1016/j.gep.2016.01.002

38. Smith, T.M., Wang, X., Zhang, W., Kulyk, W., Nazarali, A.J. (2009). Hoxa2 plays a direct role in murine palate development. Dev Dyn. 238: 2364-2373. doi:10.1002/dvdy.22040

39. Karpe, F., Pinnick, K.E. (2015). Biology of upper-body and lower-body adipose tissue--link to whole-body phenotypes. Nat Rev Endocrinol. 11: 90-100. doi:10.1038/nrendo.2014.185

40. Zha, J.P., Wang, X.Q., Di J (2020). MiR-920 promotes osteogenic differentiation of human bone mesenchymal stem cells by targeting HOXA7. J Orthop Surg Res. 15: 254. doi:10.1186/s13018-020-01775-7

41. Li, H., Huang, J., Yu, S., Li, H., Zhou, Y., Wu, Q. (2021). HOXA11-AS induces cisplatin resistance by modulating the microRNA-98/PBX3 axis in nasopharyngeal carcinoma. Oncol Lett. 21: 493. doi:10.3892/ol.2021.12754

42. Knosp, W.M., Scott, V., Bachinger, H.P., Stadler, H.S. (2004). HOXA13 regulates the expression of bone morphogenetic proteins 2 and 7 to control distal limb morphogenesis. Development. 131: 4581-4592. doi:10.1242/dev.01327
